# Rationing elective surgery for smokers and obese patients: responsibility or prognosis?

**DOI:** 10.1186/s12910-018-0272-7

**Published:** 2018-04-24

**Authors:** Virimchi Pillutla, Hannah Maslen, Julian Savulescu

**Affiliations:** 1 Monash University, Wellington Road, Clayton, VIC 3800 Australia; 2 Oxford Uehiro Centre for Practical Ethics, University of Oxford, Suite 8, Littlegate House, 16/17 St Ebbe’s Street, Oxford, OX1 1PT UK

**Keywords:** Rationing, NHS, Clinical thresholds, Personal responsibility

## Abstract

**Background:**

In the United Kingdom (UK), a number of National Health Service (NHS) Clinical Commissioning Groups (CCG) have proposed controversial measures to restrict elective surgery for patients who either smoke or are obese. Whilst the nature of these measures varies between NHS authorities, typically, patients above a certain Body Mass Index (BMI) and smokers are required to lose weight and quit smoking prior to being considered eligible for elective surgery. Patients will be supported and monitored throughout this mandatory period to ensure their clinical needs are appropriately met.

Controversy regarding such measures has primarily centred on the perceived unfairness of targeting certain health states and lifestyle choices to save public money. Concerns have also been raised in response to rhetoric from certain NHS authorities, which may be taken to imply that such measures punitively hold people responsible for behaviours affecting their health states, or simply for being in a particular health state.

**Main Body:**

In this paper, we examine the various elective surgery rationing measures presented by NHS authorities. We argue that, where obesity and smoking have significant implications for elective surgical outcomes, bearing on effectiveness, the rationing of this surgery can be justified on prognostic grounds. It is permissible to aim to maximise the benefit provided by limited resources, especially for interventions that are not urgently required. However, we identify gaps in the empirical evidence needed to conclusively demonstrate these prognostic grounds, particularly for obese patients. Furthermore, we argue that appeals to personal responsibility, both in the prospective and retrospective sense, are insufficient in justifying this particular policy.

**Conclusion:**

Given the strength of an alternative justification grounded in clinical effectiveness, rhetoric from NHS authorities should avoid explicit statements, which suggest that personal responsibility is the key justificatory basis of proposed rationing measures.

## Background

Several NHS Clinical Commissioning Groups (CCG) have recently proposed measures to restrict elective surgery for patients who either smoke or are obese. They have been described as the ‘most severe the modern NHS has seen’ and akin to ‘rationing on the basis of poverty’ due to their perceived inequity [1, 2].

The precise nature of these proposals varies between CCGs with respect to the types of surgery restricted and the specific requirements for patients and healthcare service providers. In general, patients above a certain Body Mass Index (BMI) and smokers will be required to lose weight and quit smoking prior to being considered eligible for elective surgery. Proposed time frames for what is being described as a ‘health optimisation process’ vary between 4 and 8 weeks for smoking cessation and 6–12 months for weight management. During, and at the completion of this period, patients will be clinically re-evaluated to identify whether or not they are suitable candidates for surgical intervention.

Rationing measures that restrict access to surgery from smokers and obese patients are relatively common across the NHS. Thirty four percent of CCGs have implemented at least one mandatory clinical threshold policy, which restricts access to some form of elective surgery based on either BMI level or smoking status while patients participate in health optimisation pathways [3]. However, in view of growing financial constraints, recent proposals have sought to significantly broaden the scope of such policy by expanding the nature of existing restrictions.

The Vale of York CCG has been a particularly active proponent of such policy. It presented an initial outline of its policy in September 2016, which was subsequently implemented in January 2017 following its review and approval by NHS England. Following this precedent, a number of other CCGs have sought to implement such measures, igniting public debate about whether, and if so how, the policies are justified. Whilst the NHS has argued for the need to ‘prioritise and make difficult decisions’ in the ‘current funding crisis,’ others have said that such ‘brutal service reductions’ are disproportionate and unfair [1, 4].

Within the NHS, CCGs play an important role in facilitating healthcare services for local populations. There are currently 207 CCGs, which function in partnership with local authorities in order to ‘plan, commission and performance-manage a range of local health services for their population [5].’ These include mental health, community health, emergency care, and elective hospital services. CCGs are led by an elected governing body, which consists of General Practitioners (GPs), specialist medical professionals, allied health professionals and members of the community. The aim of CCGs is to afford ‘front-line professionals more responsibility for the design of local health services’ in a manner which is sensitive to their local population’s needs [6]. In the development of proposed rationing measures, the governing bodies of CCGs consulted various stakeholders including GPs, specialist medical professionals and a cross-section of the community.

In this paper, we examine the various rationing measures presented by CCGs across the NHS. We argue that, where obesity and smoking have significant implications for the effectiveness of elective surgery, the rationing of this surgery can be justified on prognostic grounds. However, we identify gaps in the empirical evidence needed to conclusively demonstrate such effects. Furthermore, we argue that appeals to personal responsibility, both in the prospective and retrospective sense, do not provide sufficient justification for this particular policy. Accordingly, public rhetoric from NHS authorities should avoid explicit statements, which suggest that personal responsibility is the key justificatory basis of proposed rationing measures.

### Nature of proposed rationing measures

There are three primary variants of rationing policies detailing weight management and smoking cessation thresholds for elective surgery. The shared focus of each policy is a period of lifestyle intervention prior to surgery, often referred to as ‘health optimisation.’ The key variables differentiating existing policies relate to their scope, whether the policy is mandatory or voluntary, and the requirements and consequences for patients. The most controversial forms of rationing measures involve mandatory periods of health optimisation and require that patients meet specified clinical threshold criteria prior to referral for all elective surgery. Other examples are narrower in their scope, targeting only specific surgeries. Lastly, certain CCGs have adopted guidance measures in preference to enforceable clinical thresholds; these encourage voluntary patient participation in health optimisation pathways prior to surgery.

The most far-reaching examples of mandatory rationing measures enforce strict clinical threshold criteria, which restrict access to *all* forms of elective surgery on the basis of smoking status and obesity. The Vale of York CCG’s policy serves as a pertinent example of such rationing measures, having been the first to be reviewed and approved by NHS England at the end of 2016. Their policy requires patients with a BMI greater than 30 to lose 10% of their body weight or sustain a 12-month period of attempted weight loss prior to being referred for elective surgery [7]. Where significant muscle mass renders BMI measurements inaccurate, waist circumference measurements are used; a waist circumference greater than 94 cm in males and 80 cm in females mandates patient participation in health optimisation processes [7]. Similarly, any patient recorded as a smoker is required to stop smoking for 8 weeks or sustain a 6-month period of attempted smoking cessation in order to be referred for elective surgery [7]. Typically, health optimisation consists of primary care and community interventions; in more complex cases, specialist weight management and smoking cessation pathways can be utilised – the provision of these services would require the commitment of additional financial resources but would presumably be offset by the financial benefits of proposed measures, which will be discussed at a later stage. Should patients fail to adequately participate in health optimisation pathways or meet clinical threshold criteria, they will not be referred to secondary care services for elective surgical intervention – there are no further punitive measures involved. Importantly, such rationing measures contain a number of provisions to ensure patients do not experience adverse clinical outcomes either from the delay of elective surgery or as a result of participating in health optimisation. For instance, mandatory requirements only apply to non-urgent elective surgical procedures – patients requiring clinically urgent surgical procedures or surgery for diagnostic purposes are not required to meet clinical threshold criteria related to smoking and obesity and will receive surgery as required [7]. Furthermore, there are various patient exclusion criteria, which serve to accommodate clinical need and instances where patients cannot be reasonably expected to engage in and/or benefit from health optimisation. These include patients who are pregnant, have cancer or are considered ‘vulnerable,’ such as those with ‘learning disabilities, significant cognitive impairment or severe mental illness [7].’ Relevant medical professionals are also required to clinically assess patients throughout and following the completion of their engagement with health optimisation services. Therefore, assuming the effective implementation of these clinical provisions, patients should ***not*** experience clinical consequences due to proposed rationing measures. All data regarding the Vale of York CCG’s commissioning policy was retrieved from commissioning statements available on their open-access website.

Prior to the Vale of York CCG’s proposal and implementation of such broad rationing measures, the Royal College of Surgeons (RCS) conducted a review of existing commissioning policies across the NHS [3]. Data was obtained by sending freedom of information requests to all CCGs – 200 out of the then 209 replied [3]. As Table 1 shows, the review established that 1.5% of CCGs implement mandatory clinical threshold criteria on the basis of BMI measurements for all elective surgery, whilst 1% have similar policy addressing patients who smoke [3]. Only Luton CCG was shown to employ a policy targeting both smokers and obese patients [3]. Following the Vale of York CCG’s precedent, a number of NHS authorities have adopted similar rationing measures; these include East and North Hertfordshire CCG and Herts Valleys CCG. Importantly, NHS England has expressed clear support for the development of such policies: ‘We expect that many CCGs will be in the process of developing similar schemes and initiatives to deliver plans for 2017–19. This is something we would encourage, where plans are well developed and clinically validated [8].’ This strongly suggests that in the near future, such expansive rationing measures will become more common-place across the NHS.

**Table 1:** Percentage of Clinical Commissioning Groups with mandatory rationing policy broken down into specific surgery types [3]

	% of Clinical Commissioning Groups with Mandatory Rationing Policy					
	All Elective Surgery	Hip & Knee Surgery	Tonsillectomy	Surgery for Snoring	Hernia Repair	Varicose Veins Surgery
Weight Management	1.5%	22%	1%	6%	1.5%	3%
Smoking Cessation	1%	4%	2%	2.5%	2%	2.5%

Less restrictive forms of rationing measures have a narrower focus, only enforcing mandatory health optimisation and clinical threshold criteria for certain types of surgery. For example, Rotherham CCG currently implements clinical threshold criteria specifically limiting access to major joint surgery for obese patients. Patients with a BMI greater than 35 are required to commence 6 months of documented health optimisation prior to referral for elective surgery [9]. Specific clinical exceptions to threshold criteria centre on the presence of significant functional limitation and its impact on independence; other considerations include the temporal characteristics of clinical symptoms and whether or not delaying immediate surgical intervention will contribute to the increased technical difficulty of later procedures [9]. Patients are not subjected to further punitive measures if they fail to achieve specified clinical threshold criteria. All information regarding Rotherham CCGs commissioning policy was retrieved from commissioning statements accessible online. A number of CCGs implement similar policies for other surgeries including tonsillectomies, surgery for snoring, hernia repair and varicose veins surgery [3]. Table 1 summarises the incidence of such policies across the NHS.

Certain CCGs have favoured voluntary guidance measures in preference to enforceable clinical thresholds with respect to smoking and obesity. Guidance measures recommend weight loss and smoking cessation prior to elective surgical care; however, participation in health optimisation services is not mandatory. The aim of such policy is to educate and inform the patients about the risks of smoking and obesity, and the various primary care and community services that exist to enable lifestyle change. Since such measures are voluntary, there are no consequences for non-adherence. Table 2 summarises the incidence of voluntary guidance measures across the NHS – 23% of CCGs implement such policy with respect to obesity, whilst 15% do so for smoking [3]. Merton CCG highlights three primary concerns about mandatory clinical threshold policies. Firstly, concerns are raised regarding the efficacy of mandatory programmes rather than voluntary schemes. Secondly, there is a ‘recognition that more data is required to justify mandatory restriction [10].’ Finally, there are concerns that the socio-economic patterning of smoking and obesity may perpetuate existing health inequity and unfairly target poorer patient demographics [10]. These will be addressed in later sections of the paper.

**Table 2:** Percentage of Clinical Commissioning Groups with mandatory vs. voluntary rationing policies – note: policies for specific surgery types are considered to be individual policies [3]

% of Clinical Commissioning Groups with Mandatory vs. Voluntary Rationing Policies		
	CCGs with ≥1 Mandatory Rationing Policy	CCGs with ≥1 Voluntary Rationing Policy
Weight Management	31%	23%
Smoking Cessation	12%	15%

This paper will focus on the most controversial form of proposed rationing measures, which seek to enforce clinical thresholds with respect to elective surgery.

### Justification for proposed rationing measures

Debate about elective surgery rationing has centered on a variety of ethical, financial and clinical considerations. In order to assess the permissibility of the policies, we must distinguish the possible justifications for them. Certainly, growing financial pressures experienced by CCGs provide the initial impetus to more effectively distribute scarce healthcare resources. Whilst a financial justification has often been cited, it remains insufficient alone in justifying such rationing. Any rationing should also be *fair.* We argue that proposed rationing measures are primarily justified in virtue of their clinical impact on the effectiveness of elective surgery. However, there remain gaps in the empirical evidence needed to definitively demonstrate the efficacy of certain aspects of such policy.

The pursuit of financial sustainability is a key driver in the development of rationing measures targeting smokers and the obese. In 2017–18, several CCGs are expected to experience significant annualised budgetary deficits; Vale of York CCG has announced an expected cumulative forecasted deficit of £44.1 million by the end of 2017–18 [11]. The presence of such financial pressures has necessitated stricter rationing of healthcare resources. The rising prevalence of lifestyle related disease imposes a significant financial burden upon local NHS authorities. In 2006–2007, the direct cost of smoking related disease to the NHS was £3.3 billion, whilst for obesity it was £5.1 billion [12]. Accordingly, smoking and obesity have been highlighted as key areas to address in the design of cost-effective health policy. Importantly, the NHS Commissioning Board’s (2013) ‘*Ethical framework for priority setting and resource allocation*,’ dictates that the cost effectiveness of prioritisation measures must be considered in the context of their clinical effectiveness and ethical justification [13]. Therefore, whilst the scarcity of financial resources provides an argument for cutting surplus cost in *some* way, the precise way in which this is achieved – what is cut, and for whom – needs further justification.

The principal justification for proposed rationing measures targeting smokers and obese patients centres on their expected clinical benefits. Mandatory health optimisation processes are expected to positively impact health in two key ways. Firstly, such measures intend to reduce the need for surgical intervention where preoperative health optimisation has sufficient clinical effect. Secondly, that surgical intervention will be more effective due to a reduction in the incidence of perioperative complications associated with smoking and obesity. Importantly, the objective of such rationing measures is not to deny patients access to healthcare services outright or to disadvantage specific patient groups. There are numerous provisions to ensure that in circumstances where the delay of elective surgery may lead to adverse clinical consequences, patients will not be required to meet specified clinical threshold criteria. This collective clinical impact is expected to promote financial sustainability through a more efficient use of healthcare resources. Such a clinical justification relies on empirically evaluating the utility of mandatory preoperative health optimisation in addition to the relationship between smoking and obesity, and the incidence of perioperative complications.

There is a body of evidence suggesting that obesity and smoking contribute to greater perioperative complications. Patients who smoke or are obese typically experience higher rates of wound infection, pulmonary complications, cardiovascular complications, and perioperative morbidity; they also often require longer post-operative in-patient and Intensive Care Unit admission [14–18]. Importantly however, there are key differences between the efficacy of pre-operative health optimisation in reducing perioperative complications for smokers and obese patients.

There is significant evidence to suggest that pre-operative smoking cessation leads to reduced surgical complications. A 2014 Cochrane review on interventions used for pre-operative smoking cessation concluded that smoking cessation 4–8 weeks prior to surgery reduces the length of in-patient admission as well as rates of wound related, respiratory and cardiovascular complications [19]. Current evidence suggests that longer periods of pre-operative smoking cessation are more effective in reducing the incidence of post-operative complications [14, 20]. Furthermore, perioperative smoking cessation has been shown to be more effective at achieving abstinence compared to smoking cessation commenced at other time periods [19]. However, it must be acknowledged that empirical studies exploring the link between smoking cessation and perioperative benefit achieve behaviour and lifestyle change through mechanisms that do not involve mandatory participation. Therefore, whilst there is a definitive link between smoking cessation and a reduction in perioperative complications, mandatory rationing measures are only justified in virtue of expected clinical benefits provided that there is not another policy that would alter the smoking behaviour of patients in a more efficient way.

Compared to smoking, the impact of preoperative health optimisation measures for obese patients is less certain; it varies according to surgery and patient cohort. For example, a study of obese patients undergoing total joint arthroplasty found no substantial differences in readmission rates or wound related infection between patients who ‘lost or gained 5% of their body weight 1 year before their [surgery] compared to those patients who remained the same weight [21].’ However, a study of 22,367 patients undergoing bariatric surgery concluded that preoperative weight loss of approximately 10% reduces the risk of overall post-operative complications by 13–18% [22]. The ‘obesity paradox’ has been cited in attempts to explain why weight loss does not always lead to reduced perioperative complications; it suggests that to some degree, moderate obesity ‘provides metabolic reserve … that may confer [perioperative] benefit [23].’Furthermore, BMI as a measure of obesity has been increasingly challenged due to its inability to consider the distribution of adipose tissue and the presence of lean mass; both factors play a critical role within a perioperative setting [24, 25]. Therefore, whilst ‘moderate weight loss’ of 5–10% over a 6 to 12 month period is typically associated with significant clinical benefit, the paucity of empirical evidence evaluating its utility within a perioperative context undermines the clinical justification for mandatory health optimisation measures for obese patients. Until such evidence is obtained, those defending the NHS rationing polices must either have (justified) confidence that sufficient evidence will be generated (and be open to revising measures if no such evidence emerges), or must seek and defend an alternative justification (for example, grounded in the possible responsibility of obese patients for the additional costs they generate).

The principal justification for restricting elective surgery for smokers and obese patients therefore centres on their expected clinical impact. Regardless of what principles of distributive justice *should* govern the allocation of healthcare resources, the effective use of resources will remain relevant. Any plausible policy – whether based on utilitarian, egalitarian or prioritarian principles – is sensitive to whether resources are generating health improvements for those receiving them. It is not necessary that a system adopt a maximising approach to effectiveness (such as the quality-adjusted life year-maximisation approach) for effectiveness to be relevant. So, although we do not commit to a particular principle of distributive justice in this paper, we posit that the effective use of healthcare resources in a manner that considers prognostic benefit, is relevant for any healthcare system – particularly for the NHS, where financial and healthcare resources are scarce.

Some may argue that policies that focus on expected benefits are harsh and unfair towards those who cannot reasonably be expected to meet clinical threshold criteria - in particular, to patients, who for social, economic or clinical reasons, have a reduced ability to successfully engage in health optimisation pathways. Indeed, most would agree that there are considerations in addition to increasing expected benefits that are pertinent to realising a just distribution. Such considerations are not ignored by the NHS policies: there are a number of clinical exception criteria (described in the previous section), which safe-guard vulnerable patient groups. Included in these provisions is a mandate for healthcare service providers to take an active role in providing support and ongoing clinical assessment for all patients involved. This ensures continuity of patient care and prevents adverse clinical consequences. Where patients require surgical intervention in a time-sensitive manner or where they are unable to meaningfully participate in health optimisation, access to treatment will not be withheld. The purpose of such rationing measures is not to deny patients access to healthcare services outright. It is to, where possible and reasonable, more efficiently use healthcare resources through positive lifestyle and behaviour change.

Importantly, such provisions are also consonant with a focus on efficiency. The delay or denial of elective surgery for vulnerable patient groups, where the chance for successful pre-operative health optimisation is small, can lead to worsening clinical outcomes. This may require a more significant, and therefore inefficient, downstream use of healthcare and financial resources. The same applies for those patients who have sustained a 6 or 12 month period of attempted health optimisation but have failed to satisfy specified clinical threshold criteria. In these instances, patients are subsequently referred to elective surgery because an assumption is made that further investment in health optimisation services is unlikely to be sufficiently offset by gains in efficiency relating to reduced perioperative complications. Therefore, from an efficiency *and* a fairness perspective, the exclusion of certain patient groups from clinical threshold criteria is justified.

Ultimately, the clinical efficacy of proposed rationing measures would, if achieved, lead to a reduction in the incidence of perioperative complications and in the requirements for elective surgery – this is accompanied by a reduction in the fiscal burden they collectively impose. Therefore, insofar as the financial cost of providing health optimisation services is offset by this efficiency gain, the broader goals of financial sustainability are also served.

We have argued that, although there is significant empirical evidence in support of introducing mandatory preoperative health optimisation for smokers, further evidence is needed to definitively recommend such measures for obese patients; a broader evidence base is needed with respect to evaluating the efficacy of health optimisation measures in the setting of various surgeries and patient cohorts.

Note: While such an efficiency based argument may be conceivably extended to other patient characteristics which affect the efficiency of healthcare interventions, they would require equally rigorous evaluation in terms of their fairness and empirical grounding. Further discussion related to possible patient characteristics outside of obesity and smoking status is outside the scope of this paper.

### Personal responsibility as a justification

A number of authorities have invoked personal responsibility to justify proposed rationing measures. NHS England purport that mandatory health optimisation processes allow patients ‘to take responsibility for their own health [26].’ Similarly, East Riding CCG representatives have commented that such measures ‘empower patients to take greater personal responsibility for their lifestyle choices … to ensure they can lead healthier lifestyles [27].’ However, we will argue that proposed rationing measures cannot be sufficiently justified in virtue of appeals to personal responsibility, both in the prospective and retrospective sense. Crucially, though, this will not detract from the interim conclusion above: improvements in clinical outcomes, produced by health optimisation requirements, can justify rationing measures (and, in the case of smoking, *do* justify rationing measures) *whether or not* personal responsibility can be used to justify deprioritisation. Indeed, to invoke personal responsibility where it does limited justificatory work may undermine the strength of alternative justifications, especially in the context of public debate.

The notion put forward by NHS authorities that mandatory health optimisation measures invite patients to ‘take responsibility’ intuitively appears to be consonant with a prospective sense of responsibility – according to which such measures encourage individuals to make more responsible choices in the future, to avoid the potential detrimental effects of smoking and obesity. East Riding CCG representatives argue that patients should be ‘given the skills and knowledge to take accountability for their own wellbeing to ensure they can lead healthier lifestyles [27].’ However, the prospective understanding of responsibility – in so far as it assumes concerted choosing of a healthier lifestyle – is at odds with the imposition of a rationing policy affecting patients who request elective surgery prior to making lifestyle changes: any positive effects on their health will be due to mandatory health optimisation, not a concerted choice to refrain from poor lifestyle decisions. To describe compliance with health optimisation as ‘taking responsibility’ is to overstate the room for choice that the policy allows – such compliance would not constitute ‘taking responsibility’ in a way that involves responding to reasons given by the prudential or moral value of improved health per se.

That the policy promotes ‘responsibility-taking’ in the prospective sense is more plausible when considering individuals who envisage needing elective surgery in the future, and who are therefore incentivised to make changes to their lifestyles prior to being in such a position. However, even in these cases, the language used by CCGs of ‘empowering’ patients to ‘take responsibility’ seems conceptually misplaced if contingent disincentives (i.e. delayed access to healthcare services) are central to the mechanism by which – are the reasons why – behaviour change is achieved.

Previously, some have suggested that a prospective conception of personal responsibility can be grounded in principles of expected benefits and reciprocity in the justification of similar priority-setting measures. Eli Feiring’s [28] argument can be demonstrated using the following example: Consider a situation where patient ***x*** and patient ***y*** both suffer from disease ***d***, requiring elective medical treatment. Patient ***x*** has a lifestyle, which contributes to ***d*** and reduces the effectiveness of the prescribed treatment, whilst patient ***y*** does not. Here, Feiring proposes that in order for patient ***x*** to receive medical treatment on equal terms with patient ***y***, they must sign a contract committing to supported lifestyle change [28]. If patient ***x*** does not agree to sign the contract, they will receive lower priority with respect to medical treatment. Feiring suggests that on this account, personal responsibility is forward-looking because it does not use patient ***x***’s previous lifestyle, or assess their degree of responsibility for it, in order to ration treatment [28]. Feiring argues that such a contractual arrangement is justified in two key ways. Firstly, she asserts that scarce healthcare resources should be allocated according to where they will be most effective [28]. Accordingly, rationing measures should be attentive to instances where a patient’s post-treatment lifestyle reduces the expected benefits of treatment. Secondly, as Albertsen [29] demonstrates, Feiring appeals to the principles of reciprocity; she argues that ‘when resources are limited we owe it to each other to do what we can to make medical treatment efficacious [28].’ Therefore, Feiring suggests that it matters morally should patient ***x*** fail to sign or adhere to the proposed contract, such that they can be justifiably deprioritised [28].

There are several key differences between the contractual arrangement that Feiring describes and the rationing measures proposed by various CCGs – accordingly, such a conception of prospective responsibility cannot be invoked in their justification. Most importantly, proposed rationing measures do not involve the moral assessment of a patient’s choice or failure to participate in health optimisation. The voluntary nature of Feiring’s suggested contract enables the concerted choice of patients to be taken into account. However, proposed rationing measures are mandatory - patients are *necessarily* deprioritised in order to permit a mandatory period of health optimisation and to realise the subsequent increase in the expected benefits of surgical intervention. On this account, if a patient fails to meet specified clinical threshold criteria, there is no consideration of personal responsibility; they will simply not be referred for treatment (unless there is a clinical indication or relevant provision) on grounds of diminished expected benefits.

Despite their selectively restrictive nature, health optimisation measures proposed in the UK do not require nor necessarily imply a retrospective assessment of patient responsibility. Retrospective responsibility appeals to a deeper sense of moral accountability, and any policy that restricts resources on its basis would entail a revised ‘contract’ between the NHS and its consumers; patients would be required to assume responsibility (and be assessed as morally responsible) for their healthcare choices in order to freely utilise NHS services. In contrast, proposed rationing measures do not attempt to determine the moral responsibility of obese patients and smokers, but rather, address the improvements that positive health-related behaviours generate for clinical outcomes.

Although revising the ‘contract’ between the NHS and its consumers by using assessments of personal responsibility to allocate healthcare resources may have some intuitive appeal grounded in desert and fairness, it introduces difficult ethical and practical questions. The ascription of moral responsibility relies on identifying instances where an agent is justifiably open to moral criticism for their choices and actions. Importantly, this goes beyond a more straightforward identification of the merely causal role that individuals’ behaviour often plays in developing their health states.

Broadly, three stakeholders combine to varying degrees in order to determine individual health: individual agency, our biology and the environment. Biological influences relate to the way in which genetic predisposition and innate physiological characteristics contribute to health outcomes. Environmental influences more broadly comprise the social, economic and cultural phenomena, which affect our health. These are described by the World Health Organisation as the ‘conditions, in which people are born, grow, work, live, and age, and the wider set of forces and systems shaping the conditions of daily life [30].’ Therefore, any ascription of moral responsibility must contend with these non-agential influences.

It is clear that, for smoking and obesity, both biological and environmental influences play an important role. There is a general consensus amongst researchers that genetic factors contribute between 40 and 70% to the development of obesity [31, 32]. Similarly, twin studies have shown a greater than 50% heritability of cigarette smoking [33]. The social patterning of smoking and obesity highlights the role of the environment in determining health outcomes. Lower income and education, gender, and race are associated with higher rates of obesity and smoking [34, 35]. Therefore, although individual agency certainly plays some role in smoking and obesity, it is practically implausible to quantify or meaningfully delineate the extent of its contribution alongside significant biological and environmental influences in order to determine moral responsibility. Accordingly, an allocative mechanism using a retrospective assessment of personal responsibility as a means to ration healthcare resources has the potential to be inconsistent and unjust.

Given the significance of these environmental and biological influences, some may argue that deferring elective surgery, even for efficiency based reasons, unfairly targets patients for characteristics and health states they are not morally responsible for; in particular, that it may disproportionately affect low socio-economic status (SES) groups. However, as has been previously discussed, there are numerous clinical provisions included in the rationing measures, which aim to ensure that patients do not experience adverse clinical consequences due to participation in health optimisation pathways. Deprioritisation is used as an instrument to allow pre-operative health optimisation to take place and accordingly, increase the expected clinical benefit of performing elective surgery. It is not used as a punitive tool, and if implemented effectively, should not negatively impact patients. Therefore, although there will certainly be a higher proportion of low SES patients affected by proposed rationing measures, this is in virtue of the population distribution of obesity and smoking and not a reflection of targeted discrimination.

That is not to say that the concept of personal responsibility has no place in the development of health policy within the NHS or in encouraging potential patients to think about how their lifestyle choices affect the burden placed on the healthcare system. The NHS constitution states that: ‘The NHS belongs to all of us. There are things that we can all do for ourselves and for one another to help it work effectively, and to ensure resources are used responsibly [36].’ As Harold Schmidt argues, emphasising solidarity, which he characterises as ‘the notion of giving without expectation of return,’ allows the concept of personal responsibility to be used in a meaningful yet non-punitive manner [37]. On this account, being responsible for a given health state does not ‘reduce [a] person’s claims on the solidaristic community [37].’ However, personal responsibility *can* be invoked in order to promote the importance and necessity of individual agency in improving health outcomes. For example, with respect to smoking and obesity, although environmental and biological constraints certainly diminish the role of individual agency, there may ‘remain some degree of freedom for personal action and behaviour change … perhaps even a major one [37].’ In such instances, it is helpful to draw upon personal responsibility in order to ensure individuals recognise their contribution to health outcomes. As Schmidt comments, ‘achieving good health is necessarily a co-production process, requiring both individual and social action [37]**.’** This account of personal responsibility is summarised by Schmidt as the following: ‘X has played a certain causal role in having brought about [a given health state], should recognise this, and try to avoid doing so in the future [37].’ Schmidt’s remarks seem to constitute a call for agents to endeavor to *become* (more) responsible for their health states, even if they do not always or often meet the criteria for full moral responsibility when their poor health imposes a burden on the healthcare system. As such, the proposal identifies a kind of second-order, prospective responsibility – a positive duty to make positive health related choices. In turn, this is consonant with the NHS constitution, which in addition to its overarching statement appealing to solidarity and responsibility asks that patients ‘take responsibility’ for the ‘significant contribution’ they can make to their health and wellbeing [36].’

Personal responsibility employed in this nuanced manner offers a more defensible way to account for the important role of the individual in determining health outcomes. This account of personal responsibility offers an important perspective, which can inform the philosophy of the NHS in order to motivate positive health related behaviour at an individual level. However, it does not sufficiently justify why patients in this instance should be deprioritised in virtue of their smoking status or obesity. Furthermore, to consider agents’ participation in mandatory health optimisation as a necessarily result of adopting such an account of personal responsibility is to overstate the scope for concerted choice this policy permits.

Proposed rationing measures do not require an appeal to personal responsibility in order be justified. The motivation for health optimisation and necessary deprioritisation centers on the expected clinical benefits of such measures in reducing the need for elective surgery and the incidence of perioperative complications. An account of personal responsibility, which successfully engages in the complex ethical and practical issues associated with determining moral responsibility is necessary prior to justifying rationing measures in this way. Consequently, we maintain that, since it is sufficient to do the work, a justification grounded by the effects of measures on patient prognosis is more robust than one that deprioritizes patients on the grounds of retrospective moral responsibility for health states. Accordingly, public commentary from NHS authorities associating proposed rationing measures with personal responsibility should reflect the current limitations of using such a justification.

Accordingly, public commentary from NHS authorities associating proposed rationing measures with personal responsibility should reflect the current limitations of using such a justification. Invoking personal responsibility to justify mandatory health optimisation encourages the perception that such measures are punitive in nature. This has often been at the center of criticism leveled against proposed policy, which has been labeled as ‘draconian’ and akin to ‘racial or religious discrimination’, due to its selective targeting of obese patients and smokers [38]. However, if effectively implemented, clinically and social vulnerable patients will not be disadvantaged by deprioritisation. Mandatory health optimisation measures are incompatible with a prospective sense of responsibility because they do not accommodate for concerted patient choice, nor do they require an assessment of moral responsibility in the retrospective sense. Therefore, whilst it may be helpful to draw upon personal responsibility in other healthcare contexts, we suggest that authorities avoid language implying that proposed rationing measures hold patients accountable or encourage patients to take responsibility for their health-related behaviour. This is because it introduces unnecessary controversy and detracts from the more convincing justifications grounded in clinical effectiveness.

## Conclusions

Various NHS authorities have proposed rationing measures requiring mandatory pre-operative health optimisation for patients who smoke or are obese, prior to being considered eligible for elective surgery. Such measures have been ethically contentious because they aim to balance the provision of healthcare and financial sustainability; misguided discussions of personal responsibility have further added to the perception that such measures are punitive and discriminatory.

Healthcare systems with limited resources require effective rationing criteria. The reduced allocation of public money to the NHS has made achieving fiscal efficiency an even greater imperative. However, whilst the dire financial state of local NHS authorities provides the initial motivation more effective rationing measures, such measures must be fair. Where the provision of healthcare resources does not deny services to those in need, and the provision maximises the benefit from those limited resources, fairness is achieved.

The primary justification for proposed policy rests on the clinical success of mandatory health optimisation processes in successfully reducing the requirement for elective surgery and reducing perioperative complications. The established evidence suggests there is potentially significant clinical benefit for smokers but less so for obesity. More evidence is required to substantiate the relationship between weight reduction and perioperative complications, particularly to identify which patient populations should be the focus of health optimisation measures and how these measures should be enacted.

Until such evidence is present, the scope of the proposed rationing measures must be limited for obese patients, so that rationing – on the grounds of improved clinical outcomes – does not surpass the evidence base for such improvements. In order to better defend the permissibility of rationing elective surgery for both smokers and the obese, closer attention must be paid to the clinical justification for the precise parameters of the restrictions – including justification of the length, focus and nature of mandatory health optimisation periods – as well as to the evidence demonstrating their financial consequences. Most importantly, it is important that they do not ultimately deny services to those in need.

Finally, language of personal responsibility unnecessarily detracts from the central, sufficient justification of such policy. Proposed rationing measures are incompatible with a prospective sense of responsibility and they do not, and should not, seek to retrospectively ascribe moral responsibility to obese patients or smokers. Therefore, in this instance, we suggest that public commentary from NHS authorities should avoid the suggestion that proposed measures hold patients responsible or that they are justified because they encourage patients to take responsibility for their health states.

## Abbreviations

BMI: Body Mass Index
CCG: Clinical Commissioning Group
GP: General Practitioners
NHS: National Health Service
RCS: Royal College of Surgeons
SES: Socio-economic status
